# Asymmetry, sex differences and age-related changes in the white matter in the healthy elderly: a tract-based study

**DOI:** 10.1186/1756-0500-4-378

**Published:** 2011-10-04

**Authors:** Soichiro Kitamura, Masayuki Morikawa, Kuniaki Kiuchi, Toshiaki Taoka, Masami Fukusumi, Kimihiko Kichikawa, Toshifumi Kishimoto

**Affiliations:** 1 Department of Psychiatry, Nara Medical University, Kashihara, Japan; 2 Sakai City Mental Health Center, Sakai, Japan; 3 Department of Radiology, Nara Medical University, Kashihara, Japan

**Keywords:** diffusion tensor imaging, tractography, asymmetry, sex difference, aging, white matter

## Abstract

**Background:**

Hemispherical asymmetry, sex differences and age-related changes have been reported for the human brain. Meanwhile it was still unclear the presence of the asymmetry or sex differences in the human brain occurred whether as a normal development or as consequences of any pathological changes. The aim of this study was to investigate hemispherical asymmetry, sex differences and age-related changes by using a tract-based analysis in the nerve bundles.

**Methods:**

40 healthy elderly subjects underwent magnetic resonance diffusion tensor imaging, and we calculated fractional anisotropy (FA) and apparent diffusion coefficient (ADC) values along the major white matter bundles.

**Results:**

We identified hemispherical asymmetry in the ADC values for the cingulate fasciculus in the total subject set and in males, and a sex difference in the FA values for the right uncinate fasciculus. For age-related changes, we demonstrated a significant increase in ADC values with advancing age in the right cingulum, left temporal white matter, and a significant decrease in FA values in the right superior longitudinal fasciculus.

**Conclusion:**

In this study, we found hemispherical asymmetry, sex differences and age-related changes in particular regions of the white matter in the healthy elderly. Our results suggest considering these differences can be important in imaging studies.

## Background

The hemispheres of the human brain are asymmetric in structure and function, and anatomical brain asymmetry has been studied using various methods [1-9]. A postmortem study showed asymmetry of the posterior superior temporal lobe [10]. Watkins et al. reported asymmetries of the planum temporale and the angular gyrus using voxel-based statistical analysis (VBA) [9]. Likewise, Büchel et al. reported asymmetry of the arcuate fasciculus using VBA [11]. Several studies showed neuroanatomical asymmetry in the gray matter and white matter fibers [6,12,13].

Similarly, sex is a major factor affecting human brain morphology, and sex differences in the human brain have been reported [14-16]. Schalepfer et al. reported a sex difference in the volume of the language-related gray matter cortex [15], and Szeszko et al. reported a sex difference in the frontal white matter region [16].

It was reported that cognitive functions, which comprise processing speed, episodic memory and other executive functions, tend to decline with normal aging [17]. Regional brain volume loss with normal aging is associated with poor cognitive function, and frontal white matter volume correlated with cognitive performance [18]. In the elderly, degeneration of the white matter that connects the local brain regions can be associated with the decline in cognitive performance during normal aging.

Diffusion tensor imaging (DTI) is a non-invasive method of measuring the diffusion of water molecules *in vivo*, and is able to measure the quality of the neuronal fiber bundles within white matter regions of interest (ROI) [12,19]. However, the ROI approach is limited because it cannot attribute changes to specific tracts within regions containing two or more white matter bundles [20,21]. Recently, tract-based analysis has been used to investigate white matter of interest. Tract-based analysis assembles the local diffusion tensor data into tracts using scalar metrics, such as fractional anisotropy (FA) and apparent diffusion coefficient (ADC) [22]. Thus, tract-based analysis can evaluate the specific anatomical localization of a single tract and allow measurement throughout the length of the bundles.

There have been several studies on neurodegenerative diseases that have reported structural differences in hemispherical asymmetry or sex differences [23-26]. To our knowledge, it has not been discussed whether asymmetry or sex differences in the human brain are present in the healthy elderly or are always a consequence of pathological changes.

The aim of this study was to investigate the bundles of association fibers in healthy elderly subjects using tract-based analysis, and to evaluate hemispherical asymmetry, sex differences and age-related changes. In the white matter fibers, we examined the uncinate fasciculus (UNC), cingulate fasciculus (CIG), superior longitudinal fasciculus (SLF), inferior longitudinal fasciculus (ILF) and inferior occipitofrontal fasciculus (IOFF).

## Method

### Subjects

Forty right-handed volunteers (19 males and 21 females) participated in this study. The demographic characteristics of the subjects are given in Additional File 1: Table S1. All subjects were screened for medical and psychiatric conditions by a psychiatrist. Assessment of cognitive function was carried out according to the Mini-Mental State Examination (MMSE). Subjects were excluded from enrolment if they had a history of a neurological disease, of substantial head injury or a history of major psychiatric illness. Subjects with cortical infarctions on T2-weighted images were also excluded, whereas patients with small lacunae in the white matter (fewer than five lesions on T2-weighted images) were included. This study was approved by the Ethical Review Board of Nara Medical University. Written informed consent was obtained from each of the subjects prior to their participation

### Image data acquisition

A 1.5-T clinical MRI unit (Magnetom Sonata; Siemens AG, Erlangen, Germany) was used to acquire the diffusion tensor images. Diffusion-weighted images were obtained using an echo-planar imaging (EPI) sequence (TR = 4900 ms, TE = 85 ms, b = 1000 s/mm^2^, 6-axis encoding, FOV = 230 mm, matrix = 128 × 128, slice thickness = 3 mm with no gap, averaging = 6). We obtained 50 section images, covering the whole brain. In addition, we also obtained regular structural T1-weighted (SE TR = 500, TE = 20) and T2-weighted (TSE TR = 4000, TE = 110) images.

### DTI data processing

**Figure 1:**
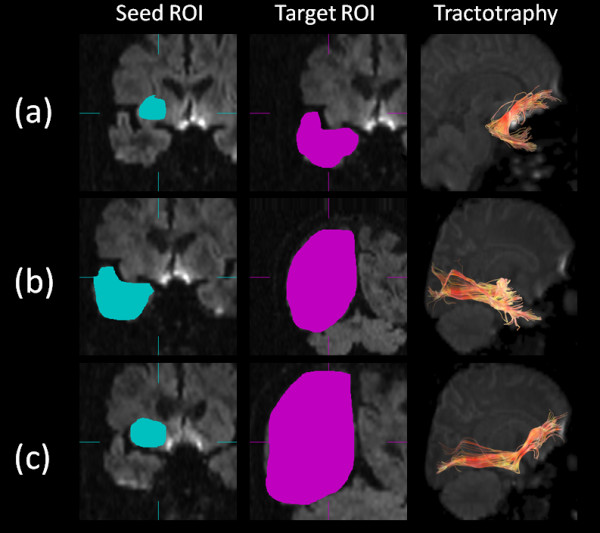
**Diffusion tensor tractographies of the (a) UNC, (b) ILF and (c) IOFF**. A light blue-colored object indicates the seed ROI, whereas a pink-colored object indicates the target ROI. UNC, uncinate fasciculus; ILF, inferior longitudinal fasciculus; IOFF, inferior occipitofrontal fasciculus; ROI, region of interest.

**Figure 2:**
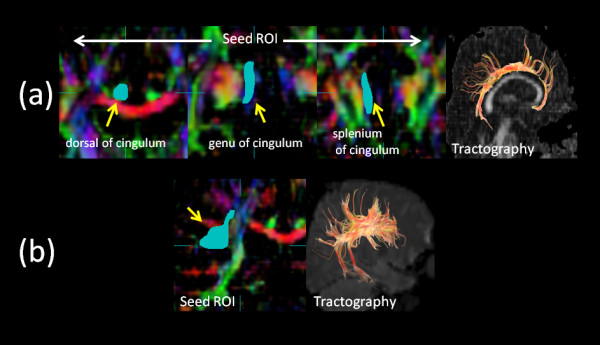
**Diffusion tensor tractographies of the (a) CIG and (b) SLF**. A light blue-colored object indicates the seed ROI. CIG, cingulate fasciculus; SLF, superior longitudinal fasciculus; ROI, region of interest.

Diffusion tensors were computed and fiber tract maps were created using Volume One and dTV II DTI software developed by Masutani et al. [27] (University of Tokyo, Diffusion Tensor Visualizer ver. 2; available at http://www.ut-radiology.umin.jp/people/masutani/dTV.htm). Interpolation along the *z*-axis was performed to obtain isotropic data (voxel size, 0.89 × 0.89 × 0.89 mm). The eigenvector associated with the largest eigenvalue or the principal axis was assumed to represent the local fiber direction. The tracking algorithm moved along the principal axis. The diffusion tensor at the next location was determined from the adjacent voxels, and its principal axis was subsequently estimated. Tracking lines were traced in this way, and propagated in both anterograde and retrograde directions until the FA fell below an assigned threshold. The settings for each tractography were as follows: the FA threshold for tracking was set at 0.18, the stop length was set at 160 steps and the seed and target ROIs are given in Additional File 2: Table S2. Using these data, we drew each fiber (Figures 1, 2). The dTV II software has a function that calculates the mean FA and mean ADC (10^-3 ^s/mm^2^) along the constructed tract. We measured mean FA and mean ADC values along the bilateral UNC, CIG, SLF, ILF and IOFF in the total subject set, in males and in females.

### Statistical analysis

Data were analyzed using the Statistical Package for Social Science (SPSS for Windows 16.0; SPSS, Chicago, Illinois). The averaged FA and ADC values of each fiber for all subjects were analyzed. Differences in measured values from the right and left hemisphere, males and females were tested using the Mann-Whitney U-test. The age-related changes were tested for statistical significance using Spearman's rank correlation analysis.

## Results

### Asymmetry

Comparisons of the mean FA and ADC values of each tractography between the bilateral hemispheres are shown in Additional File 3: Table S3. There were significant differences between each side for the ADC values of the CIG in all subjects (right, 0.395 ± 0.0124 × 10^-3 ^s/mm^2^; left, 0.402 ± 0.0174 × 10^-3 ^s/mm^2^, *p *= 0.016) and in males (right, 0.394 ± 0.0122 × 10^-3 ^s/mm^2^; left, 0.405 ± 0.0212 × 10^-3 ^s/mm^2^, *p *= 0.037). We did not observe significant differences for the CIG in females or for the other fibers.

### Sex differences

The sex differences in the mean FA and ADC values of each tractography are shown in Additional File 4: Table S4. There was a significant difference between the sexes for the FA values of the right UNC (male right, 0.375 ± 0.0100; female right, 0.366 ± 0.0136, *p *= 0.041). We did not observe significant differences for the ADC values of the right UNC or for the other fibers.

### Age-related changes

**Figure 3:**
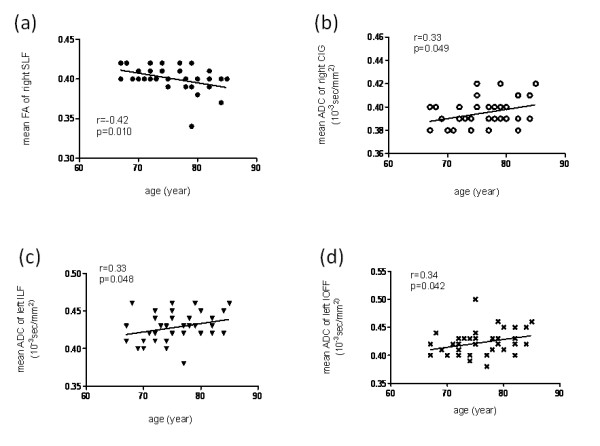
**Scatter plots and linear regression of age against (a) the mean FA of the right SLF, (b) the mean ADC of the right CIG, (c) the mean ADC of the left ILF and (d) the mean ADC of the left IOFF**. The correlation coefficients (*r*) and the *p*-values are shown. FA, fractional anisotropy; SLF, superior longitudinal fasciculus; ADC, apparent diffusion coefficient (10^-3 ^s/mm^2^); CIG, cingulate fasciculus; ILF, inferior longitudinal fasciculus; IOFF, inferior occipitofrontal fasciculus.

As regards age-related changes, we found a significant increase in ADC values with advancing age in the right CIG (*r *= 0.34, *p *= 0.034), left ILF (*r *= 0.34, *p *= 0.032) and left IOFF (*r *= 0.31, *p *= 0.045). However, there was a significant decrease in the FA values with advancing age in the right SLF (*r *= -0.39, *p *= 0.013) (Figure 3). We did not observe any significant correlations with aging for the other fibers.

## Discussion

Using a tract-based analysis, we have demonstrated hemispherical asymmetry, sex differences and age-related changes in the major white matter fibers. Tract-based analysis with DTI is considered to be a useful method for a white matter fiber assessment [28-30]. Using the conventional methods of ROI and FA mapping, it is difficult to precisely define the target because of the possibility of contamination, *i.e*., two or more white matter bundles [20,21]. Meanwhile, VBA analysis has the limitation that anatomical interpretation of the results is difficult because clusters of voxels will not lie within a single tract [31]. Using tract-based analysis, we can confirm anatomy visually, by its shape in the brain, and eliminate the effects of contamination by other white matter fibers. Simultaneously, we can measure the target bundles quantitatively.

For hemispherical asymmetry, we found a difference in the ADC values for the CIG in males and in the total subject set. Asymmetry of the white matter has been demonstrated previously using DTI analysis: Kubicki et al. reported asymmetry in the FA values of the UNC, and Peled et al. reported asymmetry in the anterior limb of the internal capsule [32-34]. In this study, we identified asymmetry of the CIG (right < left) which is consistent with a previous DTI study [12]. The CIG is known to connect the cerebral cortex and basal ganglia with the cingulate gyrus, and so this asymmetry is probably related to functional lateralization of the brain.

We also showed significantly higher FA values in males than in females for the right UNC. There are inconsistencies among studies regarding sex differences of the white matter -- some have demonstrated a sex difference, whereas others have refuted it. Hsu et al. reported that females had lower FA values than males in the right deep temporal regions, and in the left anterior limb of the internal capsule [35], which is consistent with our results. Whereas the sex difference was only gray matter proportion adjustment for the effect of cerebral volume in the large sample size study [36]. Similarly, Sullivan et al. showed no significant sex differences in the white matter using a DTI analysis [37]. However, these studies were different in methodology from our current study. Namely, the former was a volumetric analysis, and the latter was a ROI based DTI analysis. The UNC is part of the Yakovlev circuit, and connects the orbitofrontal and temporal lobes. The UNC is recognized to play an important role in the formation and retrieval of memories, and the Yakovlev circuit is related to emotion processing [22,38,39]. Our results might suggest that there is a sexual dimorphism in the microstructural organization of the white matter in the fronto-temporal region. A previous study showed the differences in emotion processing and the performance on verbal and memory tasks between males and females [38]. Thus, the sex dimorphism in this study may reflect these differences.

As regards age-related changes, we found a significant FA decrease in the right SLF and a significant ADC increase in the right CIG, left ILF and left IOFF. A previous study reported that neuronal changes in aging are due to shrinkage of large neurons with a consequent increase in the proportion of small neurons, resulting in an expansion of the extracellular space [21]. This might relate to our results. In previous studies of brain aging, Abe et al. reported that, using a voxel-based analysis, brain volume was negatively correlated with FA values in anterior structures, whereas the mean diffusivity (MD) was positively correlated with FA values in the cortical gray matter and periventricular white matter [40]. Using a tract-based analysis, Yasmin et al. reported a significant positive correlation between age and MD in the right UNC and bilateral fornices, and a negative correlation between age and FA values in the bilateral fornices [21]. Furthermore, the anterior corpus callosum, the bilateral anterior and posterior internal capsule and the posterior periventricular regions showed a significant age-related decrease in FA values [35]. The CIG is considered to play an important role in memory and cognition, the SLF is considered to relate to visual-spatial cognition, and the IOFF and ILF may relate to emotion, cognitive function and visual processing [41,42]. A decline in these cognitive functions is observed during normal aging. Namely, the changes of the DTI parameters in the CIG, SLF, IOFF and ILF may reflect this decline.

Our present study was limited by the measurement protocol for diffusion tensor imaging. We used a 6 axis diffusion encoding gradient, which is a rather small number for diffusion encoding [43]. Because the subjects of the current study was older people, the imaging time should be shorter by using smaller number of diffusion encoding. Furthermore, there is a study which indicates that number of diffusion encoding does not exert any significant effect of visualization of the optic radiation [44]. This is also the reason that the number of diffusion encoding gradients.

## Conclusions

To our knowledge, this is the first study to examine the major white matter fibers using a tract-based analysis, and simultaneously examine hemispherical asymmetry, sex differences and age-related changes. We found hemispherical asymmetry in the ADC values for the CIG in males and in the total subject set, and we identified a sex difference in the FA values for the UNC. For age-related changes, we found an increase in the ADC values for the right SLF and left IOFF, but a decrease in the FA values for the right SLF. We suggest that consideration of these local non-pathogenic changes is important when using tract-based analysis for studies of patients with psychiatric or neurodegenerative disorders.

## Abbreviations

VBA: voxel-based morphometric analysis; DTI: diffusion tensor imaging; ROI: region of interest; FA: fractional anisotropy; ADC: apparent diffusion coefficient; UNC: uncinate fasciculus; CIG: cingulate fasciculus; SLF: superior longitudinal fasciculus; ILF: inferior longitudinal fasciculus; IOFF: inferior occipitofrontal fasciculus; MMSE: Mini-Mental State Examination; MD: mean diffusivity.

## Competing interests

The authors declare that they have no competing interests.

## Authors' contributions

SK made substantial contributions to the conception of the study, had the lead in the analysis and interpretation of the data, and wrote the first draft of the manuscript. MM and KK made substantial contributions to the statistical analysis and interpretation of the data, and revised the manuscript critically for important intellectual content. TT and KK made substantial contributions to the acquisition and processing of the image data. MF made substantial contributions to the neuropsychological assessments. TK made substantial contributions to the conception and design of the study, and revised the manuscript critically for important intellectual content. All authors read and approved the final manuscript.

## Supplementary Material

Additional file 1 **Table S1**. Characteristics of the participants. Click here for file

Additional file 2 **Table S2**. Definitions of the seed and target regions of interest for each tractography. Click here for file

Additional file 3 **Table S3**. Comparisons of the right and left FA and ADC values for each tractography Click here for file

Additional file 4 **Table S4**. Comparisons of the FA and ADC values between males and females for each tractography Click here for file
